# High-Density Polyethylene–Polypropylene Blends: Examining the Relationship Between Nano/Microscale Phase Separation and Thermomechanical Properties

**DOI:** 10.3390/polym17020166

**Published:** 2025-01-10

**Authors:** Hannah Jones, Jake McClements, Dipa Ray, Michail Kalloudis, Vasileios Koutsos

**Affiliations:** 1 School of Engineering, Institute for Materials and Processes, The University of Edinburgh, Sanderson Building, King’s Buildings, Edinburgh EH9 3FB, UKdipa.roy@ed.ac.uk (D.R.); 2 School of Engineering, Newcastle University, Merz Court, Claremont Road, Newcastle Upon Tyne NE1 7RU, UK; jake.mcclements@newcastle.ac.uk; 3 Impact Laboratories Ltd. (Impact Solutions), Impact Technology Centre, Fraser Road, Kirkton Campus, Livingston EH54 7BU, UK; mkalloudis@hellenic-cables.com

**Keywords:** polypropylene, polyethylene, polymer blends, phase separation, atomic force microscopy

## Abstract

The phase separation of high-density polyethylene (HDPE)–polypropylene (PP) blends was studied using atomic force microscopy in tapping mode to obtain height and phase images. The results are compared with those from scanning electron microscopy imaging and are connected to the thermomechanical properties of the blends, characterised through differential scanning calorimetry, dynamic mechanical analysis (DMA), and tensile testing. Pure PP, as well as 10:90 and 20:80 weight ratio HDPE–PP blends, showed a homogeneous morphology, but the 25:75 HDPE–PP blends exhibited a sub-micrometre droplet-matrix structure, and the 50:50 HDPE–PP blends displayed a more complex co-continuous nano/microphase-separated structure. These complex phase separation morphologies correlate with the increased loss modulus (viscous properties) of the corresponding blends as measured by DMA, demonstrating the potential for the creation of strong and simultaneously tough, energy-absorbing materials for numerous applications.

## 1. Introduction

High-density polyethylene (HDPE) and polypropylene (PP) are inherently immiscible due to small differences in their molecular structure; however, their blends (HDPE–PP) have attracted significant interest due to cooperative effects between the two components, which create microstructures with fine-tuned mechanical properties, e.g., offering plastic components with balanced stiffness and toughness for various engineering applications [1,2,3,4,5,6,7]. Despite this immiscibility, their similar physical and chemical properties make efficient separation of their wastes challenging [8]. This is an additional reason for their widespread use as recycled blends in numerous applications requiring high strength and toughness [3,9,10,11,12].

Significant interest has been concentrated on PP-rich blends, particularly those with HDPE–PP ratios between 20:80 and 25:75, where a local maximum in regards to mechanical properties is frequently observed [13,14,15,16,17,18]. Considerable work has concentrated on the effect of the crystallinity of the two components and how one polymer impacts the crystallisation of the other [13,16,19,20,21,22,23,24,25,26]. However, the results in the published work present variability, which has been attributed to many factors, including the fabrication and processing conditions [15,26,27]. In most cases, the solidification involves rapid cooling, high shear rates, and high pressures, which impacts the overall crystallinity and results in a large proportion of the material existing in the amorphous state. Despite this, the amorphous regions are usually overlooked in morphological studies. For example, scanning electron microscopy (SEM) images of thin-film samples mainly reveal the crystalline domains in the 10–100 μm range, with phase separation deduced solely from morphological data. SEM images of fractured bulk specimens are difficult to interpret in the context of phase separation.

Atomic force microscopy (AFM), which is based on the mechanical interaction between a microfabricated nanosized tip and the interrogated material surface, has emerged as a multimode microscopic technique capable of measuring surface topography with unprecedented sub-nm resolution. Additionally, its phase imaging modes allow for the differentiation of material phases within complex material systems through measuring changes in the tip–surface interactions [28,29,30,31]. In our previous study [11], we investigated the thermomechanical properties of virgin and recycled HDPE–PP blends in a wide range of compositions. In this study, we concentrate on a subset of the virgin samples with a PP-rich content; for the first time, we present high-resolution AFM tapping mode height and phase images of the fractured samples, revealing the nano/microscale phase separation of these polymer blends. Our novel findings demonstrate the micro/nanoscale phase separation of such engineering bulk HDPE–PE blends, which is highly relevant for developing strong and tough materials for numerous applications [3,9,12]. The observed morphologies are discussed with reference to the corresponding thermomechanical data [11], which are presented again for clarity and easy comparison with the addition of a supplementary analysis of the tensile stress–strain curves (the elongation at yield and the ultimate tensile strength).

## 2. Experimental Section

### 2.1. Materials

The HDPE used (K46-06-185, Ineos, Grangemouth, UK) had a melt flow index (MFI) and density of 4.2 g/10 min and 946 kg/m^3^, respectively. The PP used (Moplen EP440G, LyondellBasell, London, UK) had a MFI and density of 1.3 g/10 min and 900 kg/m^3^, respectively. Blends of different HDPE weight percentages (wt%) in PP were used, i.e., an HDPE–PP of 0:100 (pure PP), 10:90, 20:80, 25:75, and 50:50.

### 2.2. Processing and Fabrication

PP and HDPE were received in the form of pellets. Blend fabrication was conducted using a lab-scale twin-screw extruder with a feeder (Haake MiniCTW, Karlsruhe, Germany). The conical screws had diameters of 4–15 mm, a length of 109.4 mm, and a co-rotate configuration. The extrusion process time was 5 min, with a barrel temperature of 180–185 °C and a mixing speed of 50 rpm. An injection moulder (Haake MiniJet, Karlsruhe, Germany) with a cylinder temperature of 210 °C, a mould temperature of 35 °C, an injection pressure of 50 MPa, and a hold-on pressure time of 10 s was used with the molten blends to fabricate dog-bone shaped and rectangular (for dynamic mechanical analysis) samples employing ISO 527-2-1BA [32] and 557–2296 moulds, respectively.

### 2.3. Characterisation

Differential scanning calorimetry (DSC): A Perkin Elmer DSC 8000 device (Waltham, MA, USA) was used to measure the melting and crystallisation behaviour of the HDPE–PP blends. The instrument was calibrated using an indium sample. Approximately 5–6 mg of the sample was scanned under a nitrogen atmosphere. The samples were exposed to the following thermal cycle: heating from 25 to 200 °C at 10 °C/min, isothermal treatment at 200 °C for 5 min, cooling from 200 to 25 °C at 10 °C/min, isothermal treatment at 25 °C for 2 min, and heating from 25 to 200 °C at 10 °C/min. The melting temperature, *T*_m_, and enthalpy of fusion, $∆H_{f}$, were obtained from the first heating ramp. The crystallisation temperature, *T*_c_, was taken from the cooling ramp. The degree of crystallinity was calculated using the following formula:

(1) $$\%\;\mathrm{Crystallinity}=\frac{{∆H}_{f}^{m}}{{∆H}_{f}^{p}}\times 100$$

where ${∆H}_{f}^{m}$ is the measured enthalpy of fusions for the individual PP and HDPE peaks, and the ${∆H}_{f}^{p}$ is the 100% crystalline HDPE or PP, which are 287 and 207 J/g, respectively [16]. The ${∆H}_{f}^{\;m}$ values were based on the first heating ramp, with minimal differences found when comparing the crystallinity obtained from the first and second heating ramp [11].

Dynamic mechanical analysis (DMA): The viscoelastic properties of the blends were characterised using a Tritec 2000 DMA (Triton Technology, Loughborough, UK) device in dual cantilever mode at a frequency of 1 Hz, using a temperature sweep from −50 to 150 °C at a heating rate of 5 °C/min. The sample dimensions were approximately 45 mm (l) × 10 mm (w) × 2.7 mm (d). The viscoelastic response contains an elastic component (storage modulus, *E*′) and a viscous component (loss modulus, *E*″). The glass transition (*T*_g_) and transition relaxation processes were identified as changes in the *E*″ or damping component (tan delta, $\tan\delta =\frac{E^{\prime \prime }}{E^{\prime }}$). The tan delta curve was used to estimate the *T*_g_ and other relaxation peaks present [11]. A minimum of three samples were tested, and the average and standard deviation were calculated.

Tensile testing: An Instron Tensile Machine (High Wycombe, UK), with a crosshead speed of 5 mm/min and a 10 kN load cell, was used to measure the tensile properties at ambient temperature, in accordance with the ISO 527-2 standard [32]. A Zwick Roell (Worcester, UK) Tensile Machine with a video-extensometer was used to measure Young’s moduli. The crosshead speed, gauge length, and load cell were 1 mm/min, 25 mm, and 10 kN, respectively. A minimum of five samples were tested, and the average and standard deviation were calculated.

Scanning electron microscopy: The morphologies of the virgin blends were studied using a Zeiss Crossbeam 550 FIB-SEM (Carl Zeiss Ltd., Cambridge, UK) at different magnifications. The cryo-fractured surfaces were exposed to five freeze–thaw cycles. The samples were coated with a thin layer of platinum and examined at an accelerating voltage of 3 kV.

Atomic force microscopy: AFM was conducted on the cryo-fractured PP and HDPE–PP blend surfaces using a Bruker Multimode/Nanoscope IIIa (Santa Barbara, CA, USA) in tapping mode under ambient conditions. Bruker RTESPA model probes were used for all experiments, with a nominal cantilever thickness, resonance frequency, and spring constant of 3.75 µm, 300 kHz, and 40 N/m, respectively. These probes are suitable for high-sensitivity tapping mode imaging in air; their cantilevers are aluminium-coated on the back side to increase the laser signal at the photodetector. The tips are composed of silicon (height of 15–20 µm and nominal tip radius of 8 nm) and possess a rotated shape that reduces artifacts associated with asymmetry in larger features. The height of these tips is considerably larger than any features imaged, and their sharpness ensures high image resolution. A J-scanner with a maximum x-y range of ∼160 µm was used. Both height and phase images were collected. The freeware Gwyddion, version 2.53 (http://gwyddion.net/, accessed 29 December 2024) [33] was used for image analysis.

## 3. Results and Discussion

### 3.1. Thermomechanical Properties

**Figure 1:**
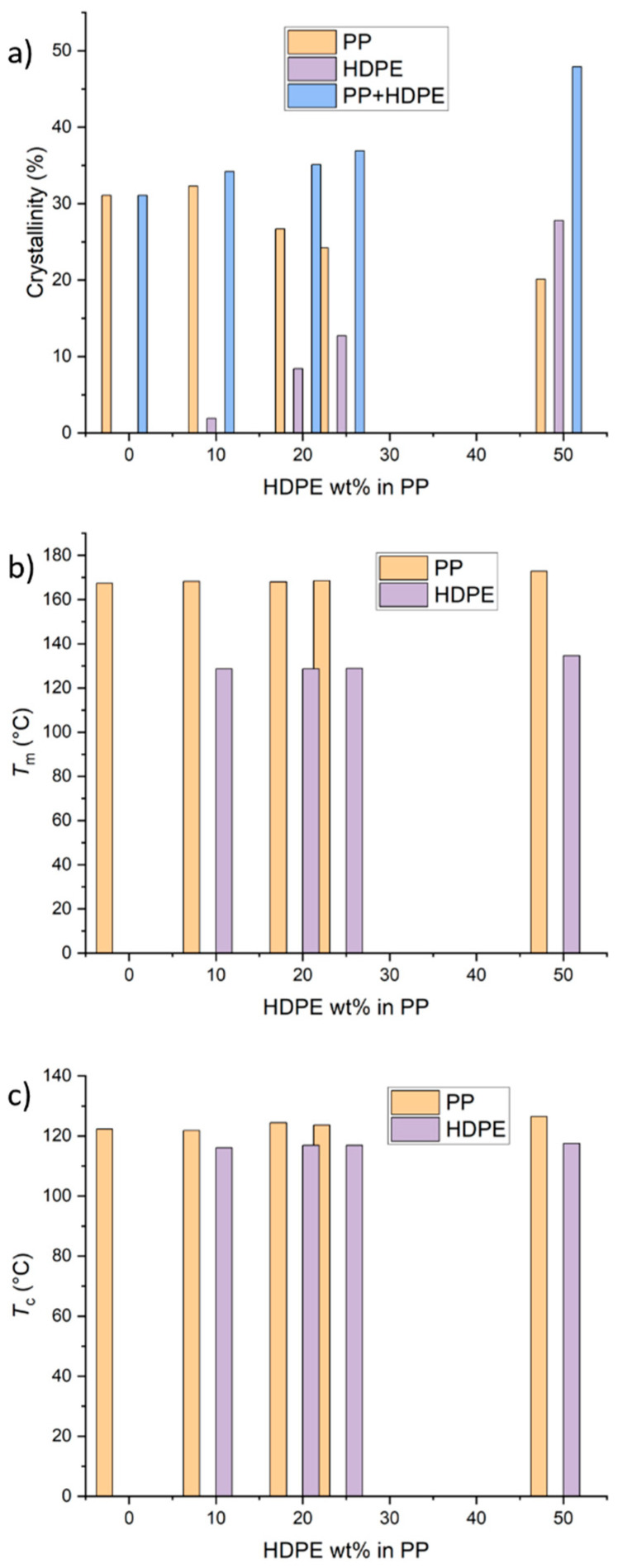
(**a**) Crystallinity, (**b**) melting temperature, and (**c**) crystallisation temperatures for pure PP and HDPE–PP blends at varying HDPE wt%.

**Figure 2:**
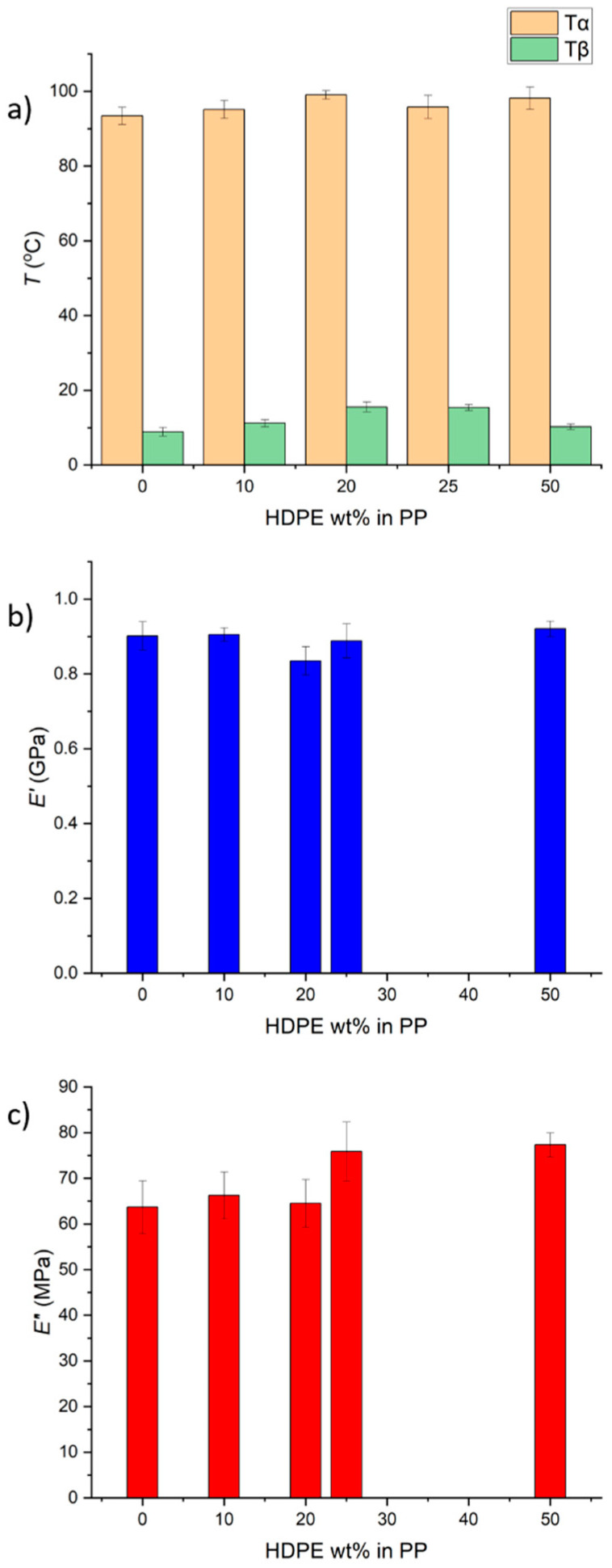
(**a**) *T*_α_ and *T*_β_ relaxation peak temperatures (taken from DMA tan delta traces), (**b**) storage modulus, and (**c**) loss modulus for pure PP and HDPE–PP blends at varying HDPE wt%.

**Figure 3:**
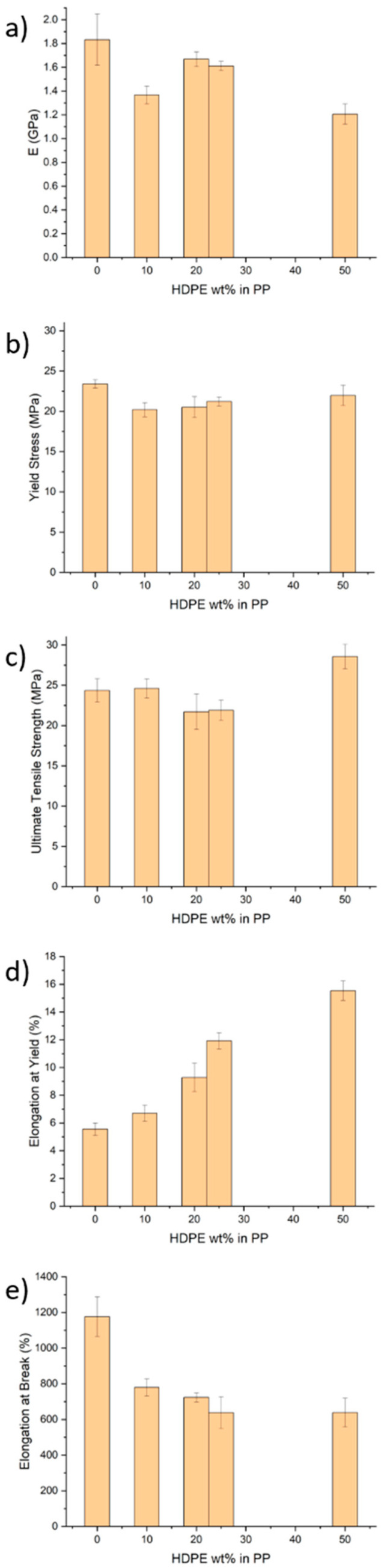
(**a**) Young’s modulus, (**b**) yield stress, (**c**) ultimate tensile strength, (**d**) elongation at yield, and (**e**) elongation at break for pure PP and HDPE–PP blends at varying HDPE wt%.

The thermomechanical properties of the PP and HDPE–PP blends, as measured by DSC, DMA, and tensile testing, are summarised in Figure 1, Figure 2, and Figure 3, respectively. The polymers are immiscible, with a maximum of 47.9% overall crystallinity at the 50:50 ratio (Figure 1a). The percentage of overall crystallinity and PE crystallinity increases with the addition of PE. However, the PP crystallinity generally decreases, reaching a minimum crystallinity of 20.1% at 50:50. The melting temperature of either polymer was not affected by the blending (Figure 1b). Similarly, minimal variation was observed in the crystallization temperatures for each polymer (Figure 1c), which indicates immiscibility and independent crystallisation behaviour.

The alpha relaxation peak temperature, *T*_α_, associated with the diffusion of defects within crystalline domains [34], is very similar for HDPE and PP and usually cannot be distinguished [11,35]. The beta relaxation peak temperature, *T*_β_, is associated with the amorphous domain branch motions and represents the *T*_g_ of PP. The *T*_β_ observed is that of PP, as the HDPE *T*_β_ is not usually distinguished due to the higher crystallinity of HDPE [11,36]. Both the *T*_α_ and *T*_β_ relaxation peak temperatures show little variation for each blend ratio (Figure 2a). Similarly, the blends exhibit minimal differences in the storage modulus, *E*′, (Figure 2b). Interestingly, the loss modulus, *E*″, for blends with higher HDPE content (25% and 50%), is approximately 20% greater than that of pure PP and blends containing 10% or 20% HDPE.

As expected, the Young’s modulus of the blends is lower than that of pure PP, but a local peak is observed at 20–25% HDPE content (Figure 3a). Interestingly, the 50:50 blend exhibits a similar yield stress to that of PP (Figure 3b) and a considerably greater (by 17%) ultimate tensile strength (Figure 3c). The elongation at yield and elongation at break display opposing trends (Figure 3d,e). This is a complicated phenomenon, as these properties are influenced by the fabrication method, particularly the injection moulding process used in this study, which can orient polymer chains in the direction of the strong flow [22,37,38,39,40]. Regardless, the elongation at break for all compositions is above 630%, while the largest elongation at yield (15.5%) occurs for the 50:50 blend, which is 180% higher than that of the pure PP.

### 3.2. Phase Morphology Analysis

The 50:50 HDPE–PP blend was initially selected as a model system to determine whether SEM could detect the sample’s phase-separated morphology. The samples were first prepared by subjecting them to a single freeze–thaw cycle before coating them with platinum. However, challenges arose during imaging due to a high level of charging, resulting in low image contrast and a lack of PP and HDPE phase clarity. To overcome this issue, the number of freeze–thaw cycles was increased to a maximum of five, based on the hypothesis that additional freeze–thaw cycles could enhance the contrast between the PP and HDPE phases due to the differing coefficients of linear thermal expansion (CLTE) [41]. The CLTE characterises the dimensional changes of a material as the temperature varies; PP has a lower CLTE than does HDPE [41], likely due to its experiencing stronger interatomic forces that limit its expansion. Despite this, increasing the number of freeze–thaw cycles did not improve phase clarity, and imaging artifacts were observed, attributed to moisture on the sample surface resulting from the freeze–thaw cycles.

**Figure 4:**
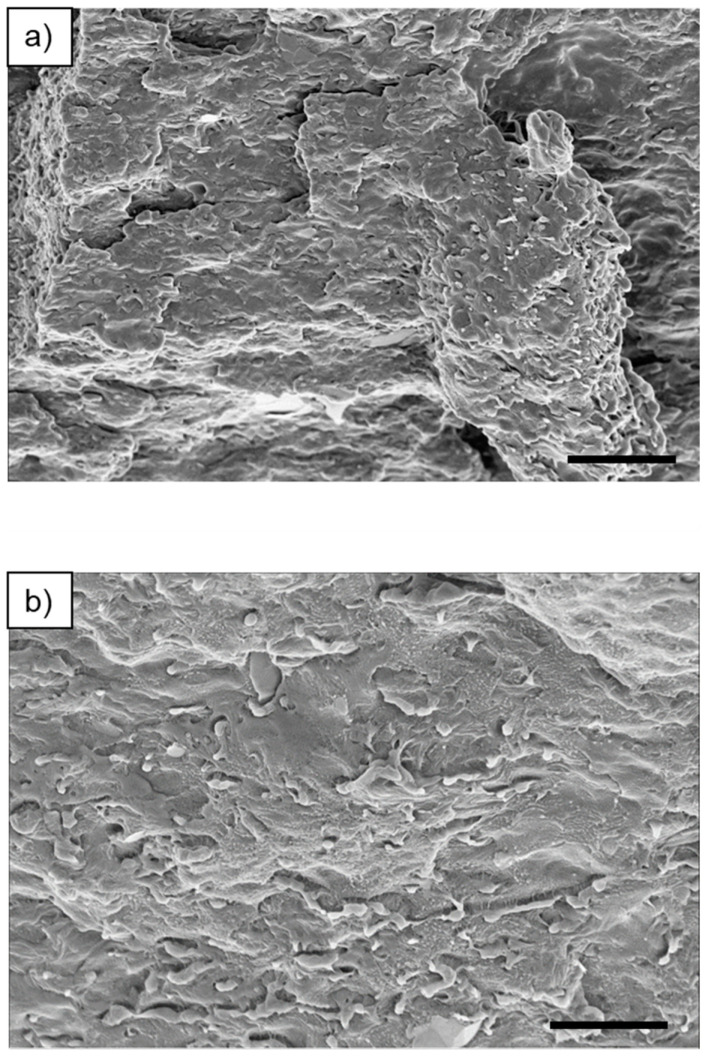
SEM images obtained for HDPE–PP 50 wt% blend: (**a**) scale bar = 5 µm; (**b**) scale bar = 2.5 µm.

SEM images showing the surface morphology of the 50:50 HDPE–PP blend are presented in Figure 4; however, the images cannot confirm whether co-continuous morphology was achieved. A similar 50:50 HDPE–PP morphology was obtained by Sutar et al. [4], who investigated a range of HDPE–PP surface morphologies using SEM. The researchers performed SEM on unfractured surfaces but could not distinguish between the HDPE and PP phases. Interestingly, Jabłońska et al. [42] found that phase clarity could be achieved for HDPE–PP blends through permanganic surface etching. This approach enabled them to deduce the overall droplet-matrix morphology and the PP and HDPE domain shapes in an 80:20 HDPE–PP blend. However, the SEM images still lacked sufficient surface phase sensitivity.

**Figure 5:**
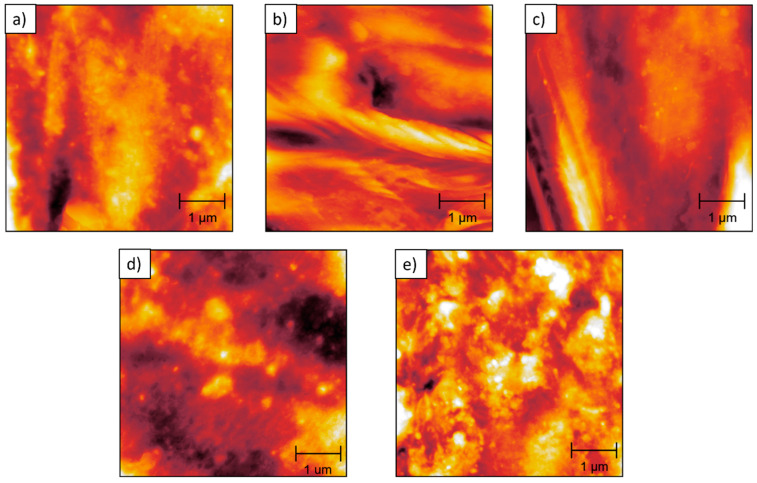
AFM height images of (**a**) pure PP, average roughness of 16.6 nm, and HDPE–PP blends at varying HDPE wt%; (**b**) 10 wt% HDPE, average roughness of 66.7 nm; (**c**) 20 wt% HDPE, average roughness of 24.9 nm; (**d**) 25 wt% HDPE, average roughness of 19.1 nm; (**e**) 50 wt% HDPE, average roughness of 14.5 nm. The height range from dark to bright colour is ca. 0–0.2 μm.

**Figure 6:**
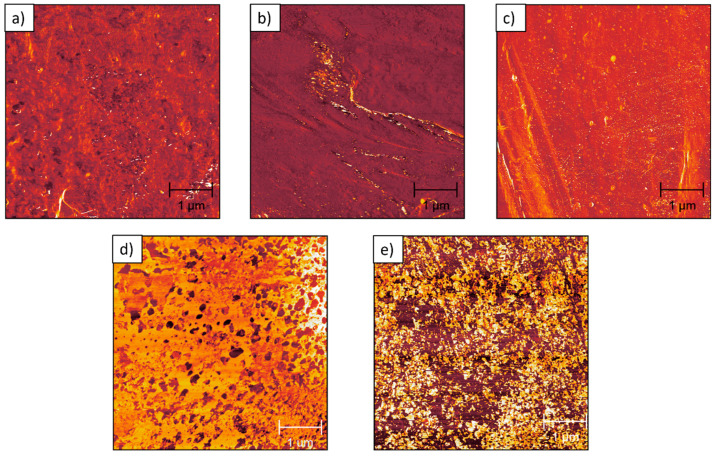
Corresponding AFM phase images of (**a**) pure PP showing a homogenous morphology and HDPE–PP blends at varying HDPE wt%: (**b**) 10 wt% HDPE, (**c**) 20 wt% HDPE, (**d**) 25 wt% HDPE, showing a droplet-matrix domain morphology, and (**e**) 50 wt% HDPE, showing a co-continuous domain morphology.

AFM height images were obtained to examine the surface morphology of pure PP and HDPE–PP blends in fine detail (Figure 5). The surface metrology properties of the polymers were calculated from these topographic images (average roughness values are provided in the Figure 5 caption). However, distinguishing between the PP and HDPE phases remained challenging. AFM phase images, which are recorded simultaneously with topographic scans, provide contrast based on differences in the phase angle of the tapping (cantilever-mounted) tip on the sample during imaging. The phase angle depends on the viscoelastic properties of a sample’s surface and therefore, can provide contrast between different materials or phases within a sample [43]. The corresponding AFM phase images of the cryo-fractured PP and HDPE–PP blends are presented in Figure 6. The phase morphology was found to be dependent upon the blend composition. A homogenous morphology was observed for pure PP, 10:90, and 20:80 HDPE–PP. Conversely, the 25:75 and 50:50 HDPE–PP blends display significant phase contrast at the sub-micron scale.

**Figure 7:**
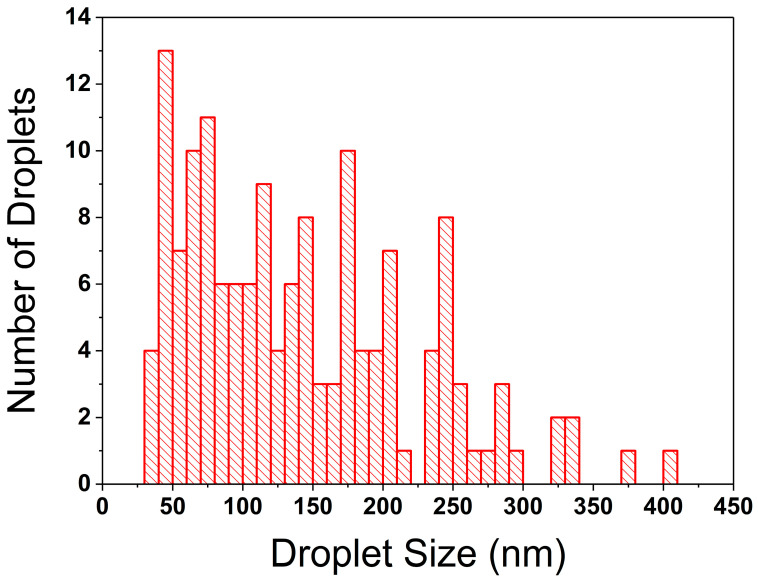
Droplet size distribution for the 25 wt% HDPE sample, taken from 150 measurements.

Within the literature, tapping mode AFM has been conducted on HDPE, PP, and HDPE–PP in binary or ternary polymer blends and composites [44,45,46,47,48]. However, there are limited investigations regarding the use of tapping mode across a range of compositions for HDPE–PP blends. As shown in Figure 6, AFM phase images of 25:75 and 50:50 HDPE–PP blends reveal a phase-separated morphology at the nano/micrometre scale. A droplet-matrix domain morphology was found at 25:75 HDPE–PP, with the HDPE dispersed in droplet domains (Figure 6d) with an average size of 142 nm, with a preference for smaller droplets (Figure 7). Moreover, a co-continuous interpenetrating nano/microscale morphology was found at 50:50 HDPE–PP (Figure 6e). Threshold analysis showed a 55% surface coverage for phase A and 45% for phase B, which is close to the expected overall 50:50 ratio of each polymer. These findings are in qualitative agreement with those of previous studies (albeit showing morphologies at a lower resolution), as Lin et al. [49] suggested a droplet-matrix structure for 30:70 HDPE–PP blends, and Jose et al. [16] found a co-continuous domain morphology from 40:60 to 60:40 HDPE–PP using SEM.

The complex morphology of the polymer blends could be related to the increase in the loss modulus (Figure 2c) for a higher HDPE content (25% and 50%) compared to that of the pure PP and blends with 10% and 20% HDPE. The droplet-matrix domain and the co-continuous domain morphology offer increased interphase areas for enhanced dissipation during deformation. Furthermore, our findings suggest that the co-continuous nano/microphase morphology is associated with increased yield stress and elongation at yield of 17% and 180%, respectively, compared to those of the pure PP, without compromising the stiffness, ultimate strength, and elongation at break (Figure 3). One possible explanation for the elongations at yield and break behaviour is that at low deformations (up to the yield point), the elongation behaviour is dictated by PP (which is less crystalline at 50:50 HDPE–PP; see Figure 1a). Once the yield point is reached, most PP chains are aligned. Consequently, HDPE cannot undergo significant necking due to its co-continuous morphology, and it breaks at lower elongations compared to those for pure PP (Figure 3e), which lacks such a co-continuous structure.

## 4. Conclusions

We have shown, for the first time, that AFM tapping mode phase imaging can visualise the phase-separated morphology of bulk engineered HDPE–PP blend samples at both the micrometre and sub-micrometre scale. This was accomplished by cryo-fracturing the samples to reveal fairly flat areas of sub-micrometre roughness that could be imaged by AFM. The observed phase-separated nano/microscale morphology was found to be dependent on the blend composition, which affected the amorphous blend parts. This signifies the importance of the amorphous part of the blends that, in many cases, constitute a high percentage of the composition but which have been largely overlooked in previous morphology studies. As the HDPE wt% increased in PP, the morphology transitioned from homogenous (pure PP, 10:90, and 20:80 HDPE–PP) to a nano/microdroplet-matrix (25:75 HDPE–PP), and finally, to co-continuous nano/microphase-separated morphology (50:50 HDPE–PP). This phase-separated morphology is associated with an increased loss modulus (viscous properties), yield stress, and elongation at yield, without compromising the other mechanical properties. Therefore, such blends offer opportunities for the development of high-toughness and high-strength materials for demanding engineering applications. Furthermore, our AFM results clearly indicate the potential of the technique for studying the phase behaviour of engineering polymer blends at the nano/microscale.

## Data Availability

The raw data supporting the conclusions of this article will be made available by the authors on request.
